# Early prediction of SIRS and sepsis development via chemiluminescent analysis

**DOI:** 10.1186/cc12920

**Published:** 2013-11-05

**Authors:** Igor V Obraztsov

**Affiliations:** 1 Department of Immunology, Federal Scientific and Clinical Center for Pediatric Haematology, Oncology and Immunology, Moscow, Russia

## Background

Neutrophils as a part of nonspecific immunity factors play a crucial role in antimicrobial resistance. Reactive oxygen species (ROS) are an important compound of the neutrophils' microbicidal action. Analysis of neutrophils' ROS production could provide valuable data on a phagocyte link of immunity [1]. A chemiluminescent (CL) assay being highly sensitive allows evaluating oxidative output of the cells in dynamics. Many studies on neutrophil CL in humans with different diseases have been published [2,3]. However, the results often vary between authors because of the lack of standardized method of CL analysis. So we have developed a methodology of neutrophils' CL analysis according to the principles of evidence-based medicine.

## Materials and methods

One hundred and twenty healthy donors and 17 ICU patients with second-third-degree burns participated in this study. We held an assay on the 1st, 8th and 15th day after injury and later; 37 observations in total. To dilute blood samples we used Hank's balanced salt saline (HBSS) with glucose, pH 7.4. Luminol (Sigma-Aldrich) was dissolved in double-distilled water at 1 mM. *N*-formyl-methionyl-leucyl-phenylalanine (FMLP; Sigma-Aldrich) and 4-phorbol-12-myristate-13-acetate (PMA; Sigma-Aldrich) were diluted in dimethyl sulfoxide (MP Biomedicals, LLC) to make stock solutions that were dissolved in HBSS on the day of experiment. CL was evaluated by means of a chemiluminometer Lum-12 (Department of Biophysics, Moscow State University) [4].

## Results

**Figure 1:**
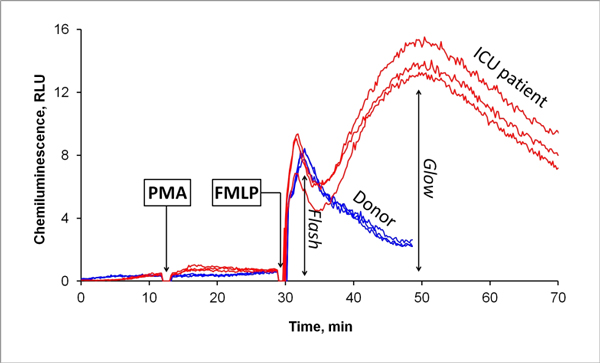
**Kinetics of CL response in ICU patient and donor**.

We substantiate an optimal experiment design in the context of obtaining the highest intensity of analytic signal and reproducible findings. Thus we have developed a method for evaluation of a neutrophil function, based on a step-by-step stimulation of the cells by PMA and FMLP. Using our approach, we investigated the distributions of CL characteristics for the population of 80 healthy donors. We obtained reproducible kinetic profiles with intensive flash and absent glow phase of emission in all of the samples. Profiles of ICU patients' samples showed high intensity of both flash and glow phase of emission (Figure 1). Insufficient glow phase indicated subsequent development of severe septic complications.

## Conclusions

As a result we suggest a reliable and replicable method for the evaluation of a neutrophil function. Investigation of the glow phase of the emission is promising to forecast risks of septic complications; we constructed a range of values of adjusted CL glow amplitude at different neutrophil counts that indicates a low probability of SIRS and septic complications that could be useful for correction of intensive treatment tactics.
